# The Coroners' Commission

**Published:** 1910-04-02

**Authors:** 


### Public Health and Hygiene.

### THE CORONERS' COMMISSION.

The Departmental Committee appointed by the Home Secretary fifteen months ago to inquire into the law and practice relating to inquests and coroners' courts, has just issued its report (Cd. 5004). The following are the main conclusions of the Committee on points of interest to medical men:—

The Committee recommends that the present rule, which requires that a county coroner should hold land, be abolished, and that in future no one who is not a barrister, or a qualified medical man, or a solicitor be appointed to the office of coroner. To this recommendation is added the conditional rider that an exception should be made in the case of a deputy who, although not a qualified lawyer or medical practitioner, has held the office of deputy for several years, and has done good service in that capacity. The Committee advocates compulsory retirement in the case of all coroners at the age of 65 years, and the granting of a pension to such coroners as have devoted all their time to the work.

### Fees and Post-mortems.

The coroner should possess the right and power to order and pay for post-mortem examinations, without an inquest, in cases of sudden death where the cause of death is obscure or unknown, but where there is no reason to suspect violence or any unnatural cause. This recommendation is made because the Committee is quite unanimous in admitting that inquests under the present system often cause a great deal of needless worry and inconvenience to relatives and friends of the deceased. The fees allowed to medical witnesses are inadequate: especially so in the case of post-mortem examination, where only a guinea is allowed as extra fee. Coroners should be empowered to call whatever expert medical evidence they consider necessary for the elucidation of the case, and to pay for such evidence at their discretion. The Committee also advises the repeal of the rule that the medical attendant of a person dying in a charitable institution should have no fee for giving evidence or for making a post-mortem examination, and fails to see why a man who gives his services out of charity should be penalised for so doing.

The Committee advocates several changes in the present system so far as juries are concerned, and points out that at present the verdicts given are sometimes bordering on the ludicrous. Before the Committee on Death Certification in 1893 it was stated that in one case the jury had found "That the deceased died from stone in the kidney which stone he had swallowed while lying on a path in a state of drunkenness," while in another case the verdict was "A child, three months old, found dead, but no evidence to show whether it had been born alive or not." Viewing the body, as at present carried out—that is to say, in a merely perfunctory manner which is quite futile—should be done away with, but in special cases the coroner should have the power to require jurors to inspect the body. The verdict of *felo de se* should be abolished. The Committee cannot imagine that the fear of such a verdict can have any deterrent effect upon persons contemplating suicide, and the only effect of retaining this verdict is to induce juries to find a verdict of temporary insanity without any evidence whatever to justify it. This latter verdict is open to the objection that when a man is dead it is impossible to say whether his insanity was of a temporary nature or not. Therefore, in cases of suicide the verdict should simply be that the defendant died by his own hand, the manner of such death being stated, but the jury should be allowed to add, if they like to do so, whether or not there is evidence to show that at the time of his death the deceased was of unsound mind.

The Committee points out that publicity is necessary and desirable in cases where the finding of the jury is a matter of great public importance. The hairwash case, in which a customer died through the use of a dangerous hairwash (tetrachloride of carbon) is cited as a case in point. On the other hand, it is evident that in many cases a public inquest often means great trouble and intense distress to the family of the deceased. Private inquiry, on the model of the Scottish system, where the procurator fiscal has power to investigate cases of sudden death in a private manner, and where a public inquest is only obligatory in cases of industrial accidents or deaths in prison, has much to recommend it, but the Committee thinks that the jury system is so deeply rooted in English life and history that there is no possibility to advocate any change. The Committee does not think that the coroner's court should be made an open court by statute, as has been suggested, but advocates a continuance of the present system whereby the coroner is allowed to clear the court if he thinks fit to do so.

### Death Certification.

Although the Committee was not specifically appointed to consider this question it was only inevitable that the problems of the present system should come before it, and that a paragraph in the report should be devoted to it. The report draws attention to the laxity of the present system and proceeds as follows:—

> One witness suggested that there was reason to fear that under the present lax system a certain number of persons might be buried alive, but we are thankful to say we have not had brought before us any authentic cases to bear out this suggestion. Mr. Pepper's evidence on this subject (twelfth day, Q. 4488 *et seq.*) was distinctly reassuring. Still, it is no fault of the law if premature burials do not take place. The present law of death certification offers every opportunity for premature burial and every facility for the concealment of crime. . . . A certificate of death should not be accepted from a medical practitioner unless it states that he has, by personal inspection of the body, satisfied himself as to the fact of death. At present he may certify merely on the information given by the relatives, and we have had evidence that many certificates have been carelessly, or even recklessly, given. Secondly, we think that in every case in which a medical certificate is not given the death ought to be reported to the coroner. It does not follow that the coroner would hold an inquest.

